# Different grassland managements significantly change carbon fluxes in an alpine meadow

**DOI:** 10.3389/fpls.2022.1000558

**Published:** 2022-10-13

**Authors:** Ganjun Xu, Xiaoming Kang, Wei Li, Yong Li, Yongyu Chai, Shengyi Wu, Xiaodong Zhang, Zhongqing Yan, Enze Kang, Ao Yang, Yuechuan Niu, Xiaodong Wang, Liang Yan

**Affiliations:** ^1^ Institute of Northwest Surveying and Planning, National Forestry and Grassland Administration, Xi’an, China; ^2^ Wetland Research Center, Institute of Ecological Conservation and Restoration, Chinese Academy of Forestry, Beijing, China; ^3^ Sichuan Zoige Wetland Ecosystem Research Station, Tibetan Autonomous Prefecture of Aba, Sichuan, China; ^4^ Beijing Key Laboratory of Wetland Services and Restoration, Chinese Academy of Forestry, Beijing, China; ^5^ Institute of Botany, Chinese Academy of Sciences, Beijing, China; ^6^ University of Chinese Academy of Sciences, Beijing, China; ^7^ College of Life Sciences, University of the Chinese Academy of Sciences, Beijing, China

**Keywords:** alpine meadow, grazing, enclosure, artificial grass planting, carbon fluxes

## Abstract

Alpine meadow plays vital roles in regional animal husbandry and the ecological environment. However, different grassland managements affect the structure and function of the alpine meadow. In this study, we selected three typical grassland managements including free grazing, enclosure, and artificial grass planting and conducted a field survey to study the effects of grassland managements on carbon fluxes in an alpine meadow. The carbon fluxes were observed by static chamber and environmental factors including vegetation and soil characteristics were measured simultaneously. Our results show that the alpine meadow was a CO_2_ and CH_4_ sink, and grassland managements had a significant effect on all CO_2_ fluxes, including gross ecosystem production (GEP, *P*< 0.001), net ecosystem production (NEP, *P*< 0.001) and ecosystem respiration (ER, *P*< 0.001) but had no significant effect on CH_4_ fluxes (*P* > 0.05). The ranking of GEP under the different grassland managements was enclosure > free grazing > artificial grass planting. Furthermore, NEP and ER at enclosure plots were significantly higher than those of the free grazing and artificial grass planting plots. In addition, different grassland managements also affected the vegetation and soil characteristics of the alpine meadow. The aboveground biomass of artificial grass planting was significantly higher than that of the free grazing and enclosure plots. The vegetation coverage under three different grassland managements was ranked in the order of enclosure > artificial grass planting > free grazing and significant differences were observed among them. Moreover, significant differences in the number of species (*P*< 0.01) and the Margalef richness index (*P*< 0.05) were detected under three different grassland managements. Further analysis of the relationship between environmental factors and carbon fluxes revealed that GEP and NEP of the alpine meadow were positively correlated with vegetation coverage, the number of species, and the Margalef richness index. Therefore, grassland restoration should be configured with multiple species, which could improve carbon sink capacity while considering the functions of grassland restoration and production.

## 1 Introduction

Grassland accounts for about one-quarter of the total land areas and is the largest terrestrial ecosystem on earth. In China, 41.7% of the territory area is covered by grassland. Grassland not only provides abundant meat, milk, fur, and other products, but also has important ecological functions, such as regulating climate, conserving water, and sequestering carbon (Wang and Wang, 2019; Bai et al., 2020). As a sensitive and fragile area to global change, the Qinghai-Tibetan Plateau plays a key role in the regional and global carbon cycle (Yang W. et al., 2021). The alpine meadow is not only the main vegetation type on the Qinghai-Tibetan Plateau but also the main carbon sink for the Qinghai-Tibetan grassland (Yan et al., 2015). As an important ecological barrier and grassland resource in China, alpine meadow has important strategic significance in the development of regional animal husbandry and ecology (Bai et al., 2018; Wang et al., 2022).

In recent decades, alpine meadows had been degraded seriously with climate change and increases in human activities, resulting in decreased biodiversity and productivity, which has affected the production and ecological function of the alpine meadow (Sun et al., 2019; Yan et al., 2020; Yuan et al., 2020; Xu et al., 2021b; Pei et al., 2022). Thus, China promulgated the Grassland Law of the People’s Republic of China and implemented a policy of banning grazing by fencing and successively returning grazed land to grassland. Fence enclosure (hereinafter referred to as enclosure) and artificial grass planting are widely used to restore degraded grasslands. Enclosure is a natural renewal and restoration method for grasslands by fencing a fixed area of grass so that it is undisturbed by human activities, such as grazing (Wang et al., 2019; Sun et al., 2020). Artificial grass planting involves establishing annual or perennial artificial grass in a fence (Bai et al., 2018). Under these grassland managements, the grassland degradation had been contained.

Studies have shown that plant biomass, vegetation carbon, and ecosystem carbon stocks significantly increased after 3 and 16 years of artificial grassland planting in a degraded alpine meadow, whereas soil respiration rates during the growing season, non-growing season, and the whole year do not change significantly. Artificial grasslands have a strong carbon sink potential and are suitable for restoring the alpine meadows of the Qinghai-Tibetan Plateau (Li et al., 2018; Huang et al., 2022). However, some studies have reported that the species composition of artificial grasslands is relatively simple, which reduces soil multifunctionality in the alpine grassland (Chen et al., 2021; Xu et al., 2021a). A 3-year controlled experiment revealed that although artificial grasslands have higher coverage and soil carbon, naturally restored plots have more species, higher biomass, and a higher root-shoot ratio. Artificial grassland planting is not necessarily better than enclosure in extremely degraded alpine meadows when considering plant diversity and soil nutrients (Tang et al., 2021). Due to the rapid restoration of vegetation by artificial grass planting of only one or a few species, low biodiversity and poor community stability may lead to more serious degradation and a waste of human and material resources during recovery. On the other hand, a close-to-nature restoration of alpine grasslands leads to higher biodiversity and ecosystem multifunctionality after enclosure; thus, has higher resilience to disturbances (He et al., 2020). A field study of Haibei alpine meadow reported that artificial perennial grasslands produce a large amount of high-quality forage and maintain the soil carbon pool simultaneously. In contrast, it’s no output of livestock products in the enclosure plot. Therefore, artificial perennial grassland could be considered in the perspective of both production and ecological functions (Zhang Z. et al., 2018). In all, grassland ecosystem that recovers naturally after enclosure is stable, but the recovery process is long. Artificial grass planting could rapidly improve vegetation coverage and biomass, but results in relatively low biodiversity and stability. Previous studies mainly focused on the aspects of diversity, stability, and grass yield. Different grassland managements not only affect stability and biodiversity but also affect the carbon fluxes (Medina-Roldán et al., 2012; Deng et al., 2017; Du and Gao, 2021). In the context of the “double carbon” goal, different grassland managements need to be compared from the perspective of carbon fluxes.

In this study, we hypothesize that grassland managements have significant impact on carbon fluxes of alpine meadow. Both enclosure and artificial grass planting has higher net ecosystem production due to the higher aboveground biomass. Specially, field survey of different grassland managements (including free grazing, enclosure, and artificial grass planting) on the carbon fluxes in a typical alpine meadow was conducted to explore (1) the effects of grassland managements on carbon fluxes, (2) the effects of grassland managements on environmental factors, (3) relationships between carbon fluxes and environmental factors with the aim to provide database and reference for recovering alpine meadow and accurately assessing the carbon sink.

## 2 Materials and methods

### 2.1 Study area and experimental design

This study was conducted in Maqin County, Guoluo Tibetan Autonomous Prefecture, Qinghai Province, China. Maqin County is located in the national key ecological protection area of the “Three River Sources”, with an average elevation of more than 4,100 m. It is a semi-humid plateau continental climate with an average annual temperature of −0.5°C and an average annual rainfall of 514 mm. It is humid and cool during the summer and dry during the winter (Yang et al., 2013; He et al., 2018). The soil types are alpine meadow soil, meadow swamp soil, and alpine shrub meadow soil (Wang et al., 2011; Gao et al., 2016).

**Figure 1:**
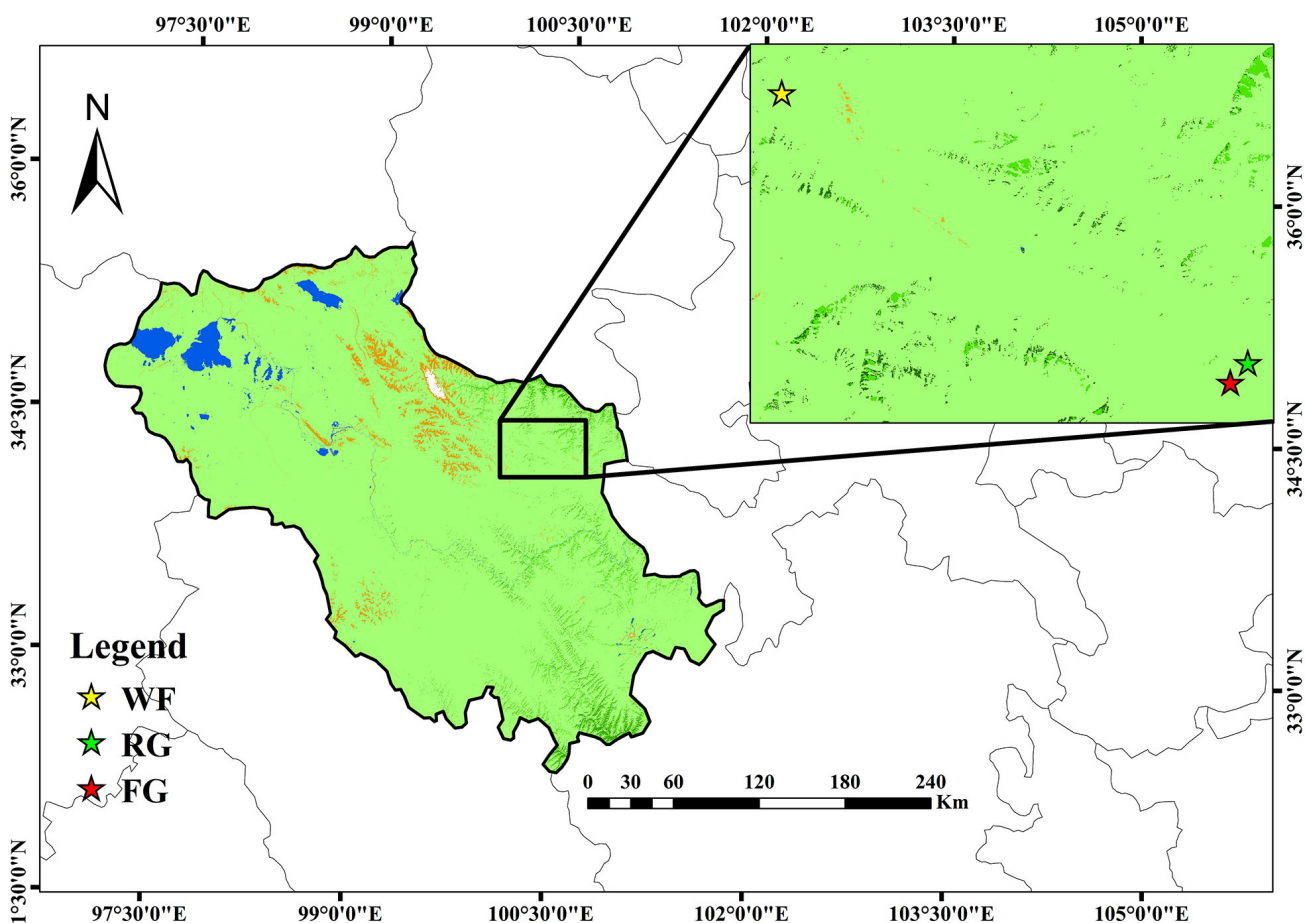
Locations of the sampling sites in Maqin County. FG, free grazing; WF, enclosure; RG, artificial grass planting.

At first, we started literature review to determine the three main grassland managements in the study area. Then we conducted an overview field survey for 3 days at Maqin by communicating with local management departments and herdsmen simultaneously. After that, we selected three sites, including artificial grass planting (RG), free grazing (FG), and enclosure (WF) (**Figure 1**). The RG site was selected from an artificial grassland established in 2015 and recovered from a severely degraded alpine meadow (100.49°E, 34.36°N), with mixed sowing of native perennial grasses *Poa pratensis L* and *Elymus nutans Griseb* at a 1:1 ratio. FG site was selected from a fenceless and public alpine meadow (100.48°E, 34.35°N). It’s grazed by yaks and with stocking capacity of 2–3 yak/hm^2^. The dominant species are *Kobresia myosuroides* and *Ajania tenuifolia.* WF site was selected from a private meadow of a herdsman (100.20°E, 34.48°N). The fences were used 3 years ago to allow vegetation to grow naturally and to prohibit grazing. The dominant species were *Ligularia virgaurea*, *Lancea tibetica*, and *Poa annua L* (**Table 1**).

**Table 1:** Dominant species of each sample site.

Grassland managements	Dominant species
Free grazing (FG)	*Kobresia myosuroides, Ajania tenuifolia*
Enclosure (WF)	*Ligularia virgaurea, Lancea tibetica, Poa annua L.*
Artificial grass planting (RG)	*Poa pratensis L., Elymus nutans Griseb., Lancea tibetica*

### 2.2 Measurement of ecosystem CO_2_ and CH_4_ fluxes

CO_2_ fluxes include net ecosystem production (NEP), ecosystem respiration (ER), and gross ecosystem production (GEP). NEP was measured under light conditions (a positive value represents carbon absorption and a negative value represents carbon emissions). Then, a shaded black cloth was measured on the static box, and the measured value was the ER. CH_4_ fluxes were measured simultaneously with NEP. The sum of ER and NEP was GEP. Ecosystem CO_2_ and CH_4_ fluxes were observed with a static chamber (50 × 50 × 50 cm) coupled with a rapid greenhouse gas analyzer (GLA132-GGA, ABB, Edmonton, AB, Canada). The closed static box was made of an aluminum alloy skeleton and a transparent acrylic plate. The data acquisition frequency of the analyzer was 1 Hz. Flux measurements were carried out from 9:00 a.m. to 2:00 p.m. on clear and cloudless days (Zhang T. et al., 2018). Three typical quadrats were selected to insert a 50 cm × 50 cm stainless steel metal base into the soil at the RG, FG, and WF sites. The static box was placed on a stainless steel metal base, and there were grooves around the upper part of the base. During measurements, the water was poured into the groove to ensure that air could not get between the box and the base. There were two circular holes with diameters of 5 cm in the center of the top of the static box. Two rubber plugs were connected to the rapid greenhouse gas analyzer with a circular hole through the rubber plugs. These tubes were used to introduce and recycle gas into the greenhouse gas analyzer to measure fluxes. Two 12 V battery-powered fans were installed on the inner wall of the static chamber to ensure full gas mixing during measurements. Each chamber was measured for 2 minutes, and the instantaneous concentrations of CO_2_ and CH_4_ were determined. The air temperature in the chamber was measured by TZS-5X thermometer, whereas another thermometer was inserted into the soil at different depths to measure soil temperature (Ts).

The rates of CO_2_ and CH_4_ fluxes were calculated according to the linear slope of the gas concentration over time:


(1) $$Fc=\frac{M}{V_{0}}\times \frac{P}{P_{0}}\times \frac{T_{0}}{T_{0}+t}\times \frac{d_{c}}{d_{t}}\times H$$

Where *Fc* is gas flux (mg m^-2^ h^-1^); *M* is the molar mass of the measured gas (g mol^-1^); *dc*/*dt* is the slope of the gas concentration over time (ppm h^-1^); *V_0_* is the molar volume of gas (m^3^ mol^-1^). *T_0_* and *P_0_* represent the absolute temperature (K) and atmospheric pressure (Pa) under standard conditions, respectively. *P*, *t*, and *H* are the measured atmospheric pressure (Pa), temperature (K), and the height of the closed static box (m), respectively.

### 2.3 Measurement of environmental factors

Three 0.5 m × 0.5 m quadrats were randomly set in each grassland management site. Vegetation coverage (VC) was investigated in each quadrat. The name, number and coverage of each species were recorded for each quadrat. The aboveground biomass (AGB) in each quadrat was harvested, numbered in an envelope, and dried continuously for 72 h in a 60°C oven to constant weight. Soil samples were collected from the 0–10, 10–30, and 30–50 cm layers using a 3.8-cm diameter soil drill. The soil samples were screened using a 2-mm mesh sieve to remove stones and fine roots. Soil water content (SWC) was measured by the drying weigh method, Soil pH was measured by PHS-3C pH meter (Hangzhou Orion Instrument Co. Ltd., Hangzhou, China). We used HCl to remove inorganic carbon in soil samples, and ultrapure water to wash off HCL, then soil organic carbon (SOC) was measured by elemental analyzer. Soil total nitrogen (TN), soil total phosphorus (TP) and available phosphorus (AP) was measured by elemental analyzer, molybdenum antimony blue colorimetry, NaHCO_3_ extraction–molybdenum antimony anti-colorimetric, respectively.

### 2.4 Calculation of the community characteristic indices

The Shannon-Wiener diversity index, Pielou evenness index, and the Margalef richness index were calculated after the quadrat survey. The calculation method is:


(2) $$H=\sum_{i=1}^{s}\left[P_{i}\times ln\;(P_{i})\right]$$

(3) P=H/ln (S)

(4) M=(S−1)/ln (N)

Where *H*, *P* and *M* is the Shannon-Wiener diversity index, the Pielou evenness index, and the Margalef richness index respectively. *S* is the number of species, *N* is the total number of individuals of all species in the sample, and *P*_i_ is the proportion of individuals on the ith plant to the number of individuals of all species.

### 2.5 Statistical analysis

One-way analysis of variance and the least-significant difference test was used to detect differences in the carbon fluxes and vegetation and soil characteristics in the alpine meadow ecosystem under the RG, FG, and WF grassland managements. The correlation between each carbon flux component and vegetation and soil characteristics was analyzed. All statistical analysis and visualization were completed based on R4.0.4 platform (The R Foundation for Statistical Computing, Vienna, Austria).

## 3 Results

### 3.1 Effects of different grassland managements on carbon fluxes

**Figure 2:**
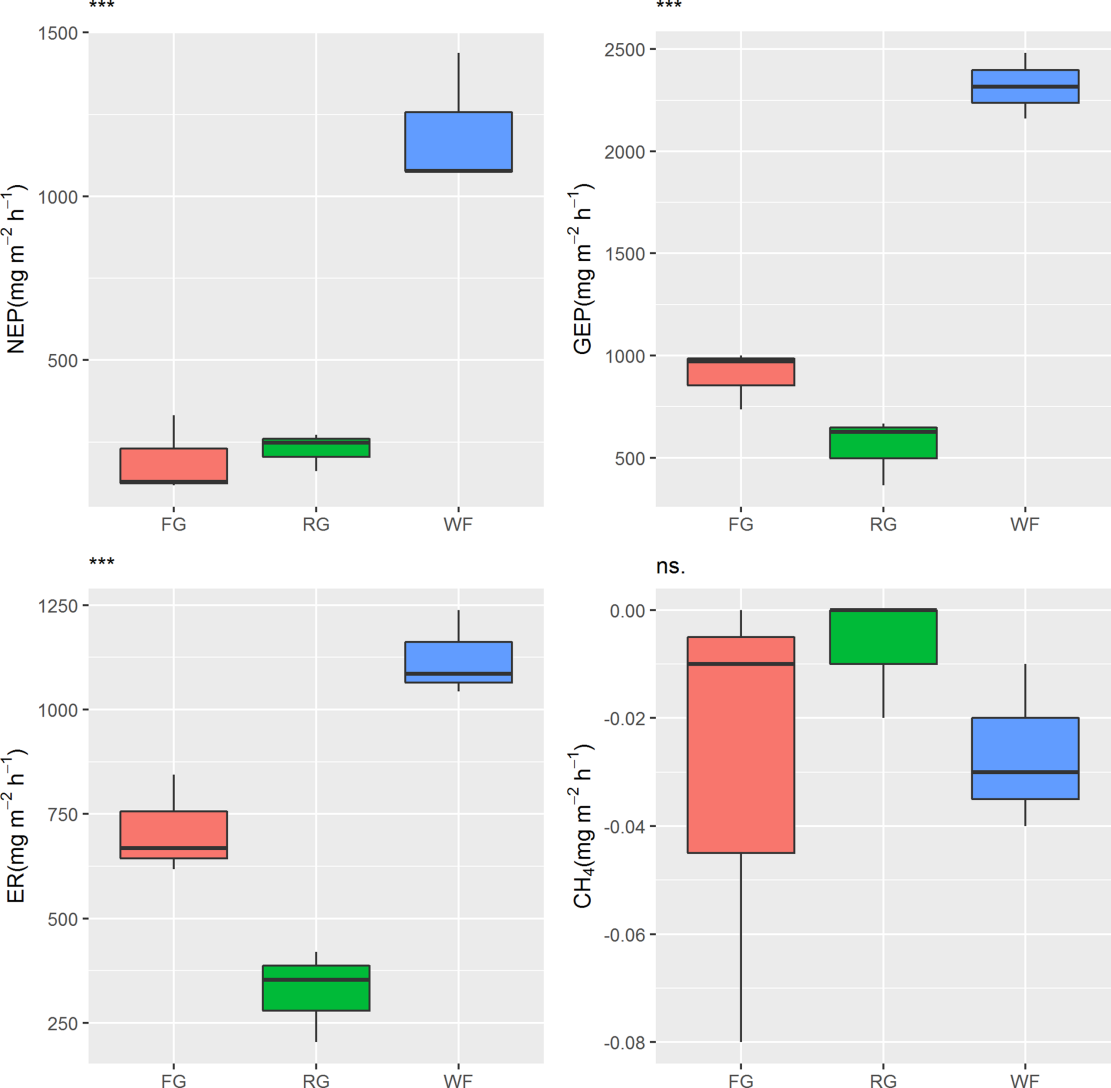
Effects of different grassland managements on carbon fluxes in the Maqin alpine meadow. NEP: net ecosystem production, GEP: gross ecosystem production, ER: ecosystem respiration, CH_4_: methane fluxes, FG: free grazing, RG: artificial grass planting, WF: enclosure; “***” and “ns.” represent significant relationships of *P*< 0.001 and *P* > 0.05, respectively.

The NEP of the alpine meadow ranged from 118.73 to 1,437.55 mg CO_2_ m^-2^ h^-1^, indicating a carbon sink. However, significant differences in NEP were observed under the different grassland managements (*P*< 0.001). The NEP (mean 1,196.22 mg CO_2_ m^-2^ h^-1^) of the WF plots was significantly higher than that of the FG and RG plots, which was 5–6 times higher than that in the FG and RG plots. No significant difference in NEP was detected between the FG and RG sites in the alpine meadow. The different management methods also had significant effects on GEP (*P*< 0.001). The rank order of GEP from large to small in the alpine meadow was WF > FG > RG (**Figure 2**). The GEP of WF was more than 2,000 mg CO_2_ m^-2^ h^-1^, and the mean value (2,318.98 mg CO_2_ m^-2^ h^-1^) was 2.6 and 4.2 times higher than that of FG (902.94 mg CO_2_ m^-2^ h^-1^) and RG (552.89 mg CO_2_ m^-2^ h^-1^), respectively. The mean ER value in the WF plots was 1,122.76 mg CO_2_ m^-2^ h^-1^, which was significantly higher than that of the FG (710.17 mg CO_2_ m^-2^ h^-1^) and RG plots (325.69 mg CO_2_ m^-2^ h^-1^). The CH_4_ fluxes of the alpine meadow were −0.08 to 0 mg CH_4·_m^-2^ h^-1^, representing a CH_4_ absorption sink, but the effect of the grassland managements on CH_4_ fluxes was not prominent (*P* > 0.05).

### 3.2 Effects of different grassland managements on environmental factors

#### 3.2.1 Effects of different grassland managements on vegetation characteristics

**Figure 3:**
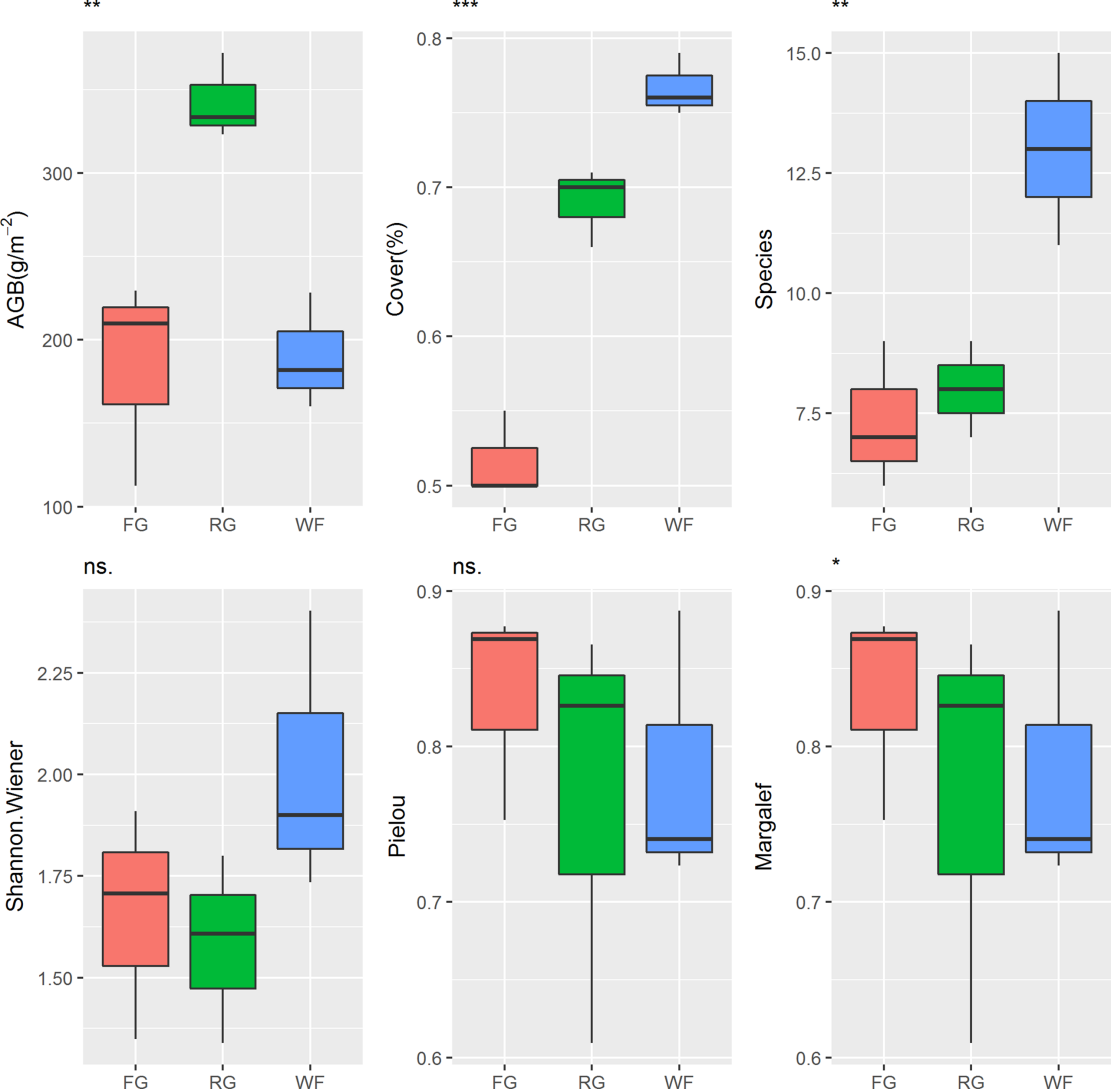
Effects of different grassland managements on vegetation characteristics at Maqin alpine meadow. AGB: aboveground biomass, Cover: vegetation coverage, Species: number of species, Shannon-wiener: Shannon-wiener index, Pielou: Pielou index, Margalef: Margalef index, FG: free grazing, RG: artificial grass planting, WF: enclosure; “***”, “**”, “*” and “ns.” represent significant relationship of *P*< 0.001, *P<*0.01, *P<*0.05 and *P* > 0.05, respectively.

Different managements had significant effects on AGB in the alpine meadow (*P*< 0.01). The AGB of the RG was significantly higher than that of the FG or WF plots (**Figure 3**). The VC of the alpine meadow was ranked in the order of WF > RG > FG and the difference among different grassland managements was significant (*P*< 0.001). No significant differences in the Shannon-Wiener index or the Pielou index were observed in the alpine meadow under the different grassland managements, but there was a significant difference in species number (*P*< 0.01) and the Margalef index (*P*< 0.05). The number of species in the WF plots ranged from 11 to 15, which was significantly higher than in the RG (7–9 species) or FG plots (6–9 species).

#### 3.2.2 Effects of different grassland managements on soil physicochemical properties

**Figure 4:**
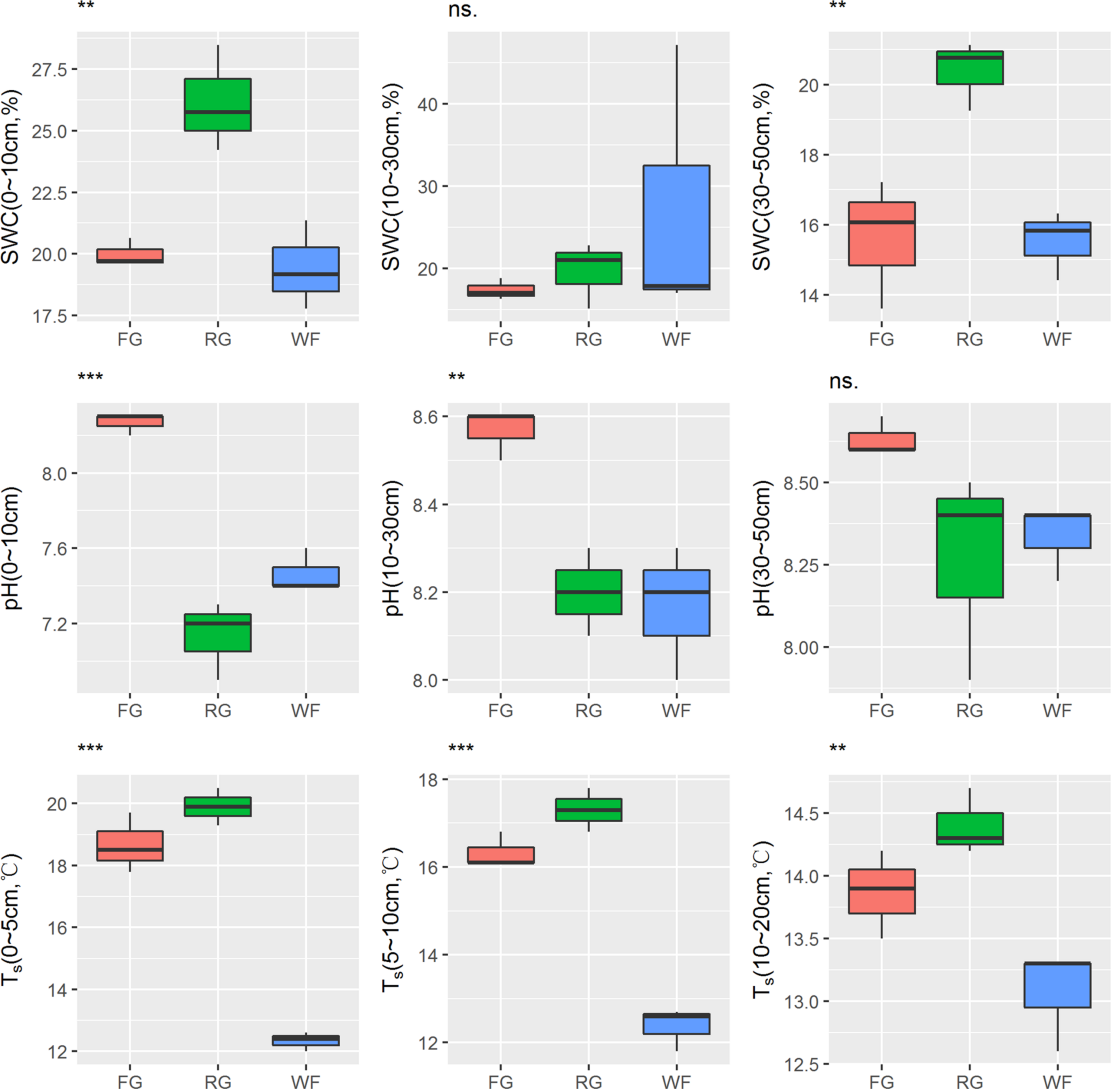
Effects of different grassland managements on soil water content (SWC), pH and soil temperature (Ts) at Maqin alpine meadow. FG: free grazing, RG: artificial grass planting, WF: enclosure; “***”, “**”and “ns.” represent significant relationship of *P*< 0.001, *P*< 0.01 and *P* > 0.05, respectively.

The soil moisture content of the alpine meadow was different depending on the grassland managements and soil depth. SWC in the 0–10 cm soil layer of RG was significantly higher than that of WF or FG (*P*< 0.01). No significant difference in SWC of the middle soil layer (10–30 cm) was detected under different managements. SWC in the 30–50 cm soil layer of RG plots was significantly higher than that of WF and FG plots. Moreover, soil pH of different layers was significantly higher in the FG plots than in the WF and RG plots. The soil temperature in each soil layer was significantly lower in the WF plots than that in the FG and RG plots (**Figure 4**).

**Figure 5:**
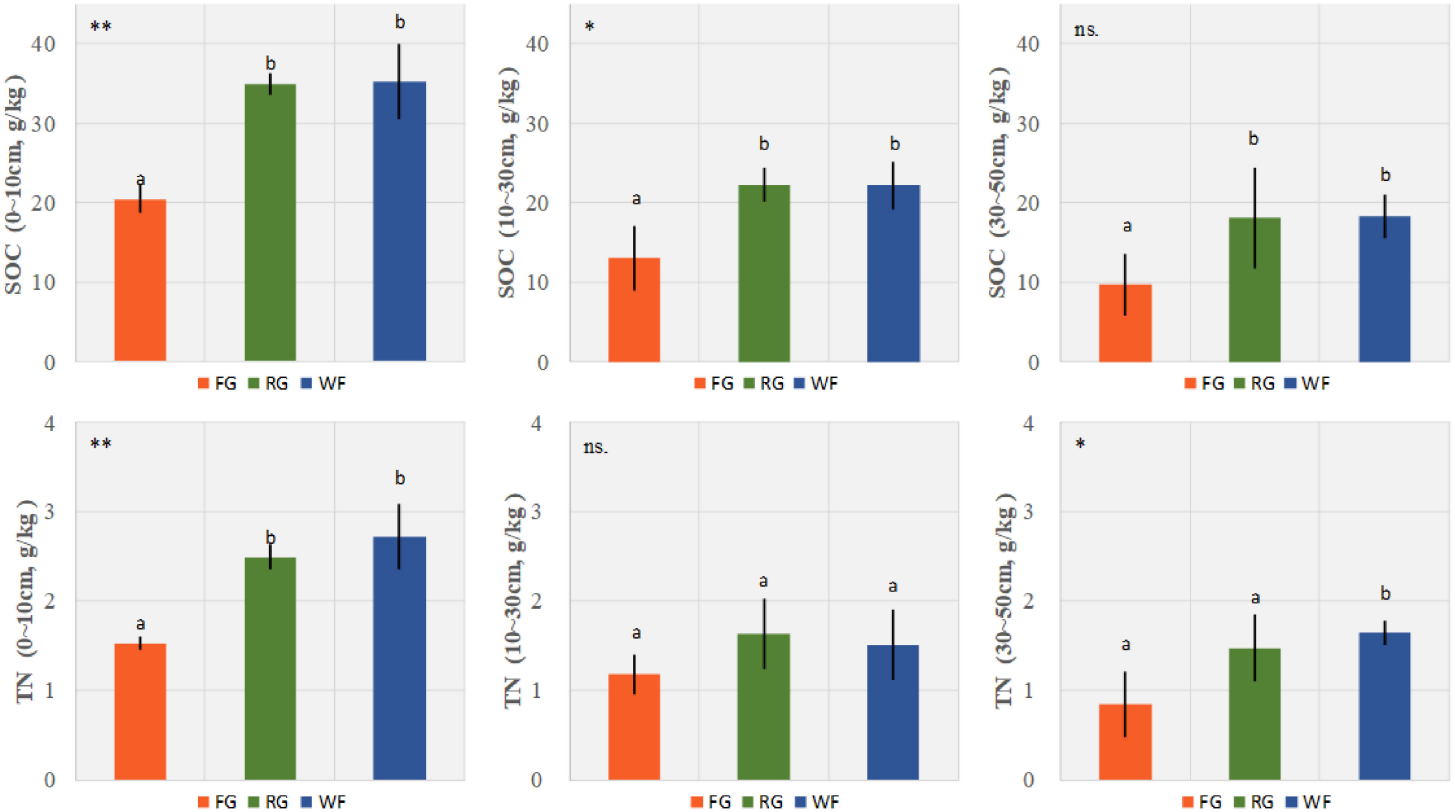
Effects of different grassland managements on soil organic carbon (SOC, mean ± SD) and total nitrogen (TN, mean ± SD) at Maqin alpine meadow. FG: free grazing, RG: artificial grass planting, WF: enclosure; “**”, “*” and “ns.” represent significant relationship of *P*< 0.01, *P*< 0.05 and *P* > 0.05, respectively. Different letters above histogram indicate significant differences (*P*< 0.05) between two grassland managements.

Different grassland managements had significant effects on SOC of the surface (0–10 cm, *P*< 0.01) and middle (10–30 cm, *P*< 0.05) soil layers. The SOC levels of the WF and RG plots were significantly higher than those of the FG plots, but no significant difference was detected between the WF and RG plots. SOC of the 30–50 cm layer was significantly higher in the WF plots than the FG plots. Furthermore, the grassland managements had a significant effect on TN except in the middle layer (10–30 cm). The TN contents of the surface (0–10 cm, *P*< 0.01) and deep (30–50 cm, *P*< 0.05) soil layers of the WF plots were significantly higher than those of the FG plots, but no significant difference was observed between the WF and RG plots (**Figure 5**). No significant difference was observed for TN in the middle (10–30 cm) soil layer.

**Figure 6:**
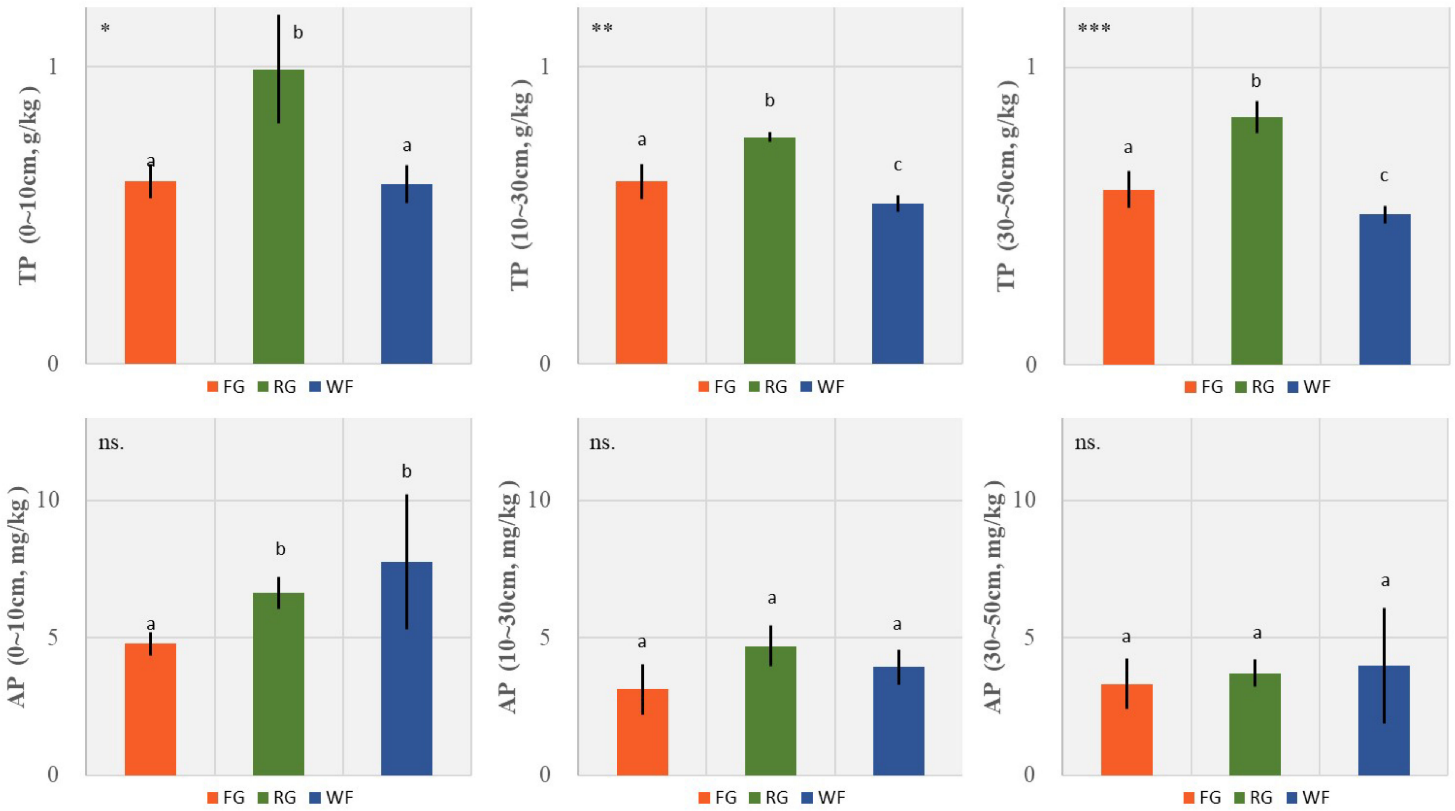
Effects of different grassland managements on soil total phosphorus (TP, mean ± SD) and available phosphorus (AP, mean ± SD) at Maqin alpine meadow. FG: free grazing, RG: artificial grass planting, WF: enclosure; “***”, “**”, “*” and “ns.” represent significant relationship of *P*< 0.001, *P*< 0.01, *P*< 0.05 and *P* > 0.05, respectively. Different letters above histogram indicate significant differences (*P*< 0.05) between two grassland managements.

The TP content of the RG plots was significantly higher than that of the WF and FG plots. No significant difference was detected between surface TP (0–10 cm) of the FG and WF plots. The TP content in the middle (10–30 cm) and deep (30–50 cm) soil layers were ranked in the order of RG > FG > WF. However, soil AP in the surface layer (0–10 cm) of the FG plots was significantly lower than that in the WF and RG plots, and no significant difference was observed between the middle and deep soil layers among the three managements (**Figure 6**).

### 3.3 Relationships between carbon fluxes and environmental factors

**Figure 7:**
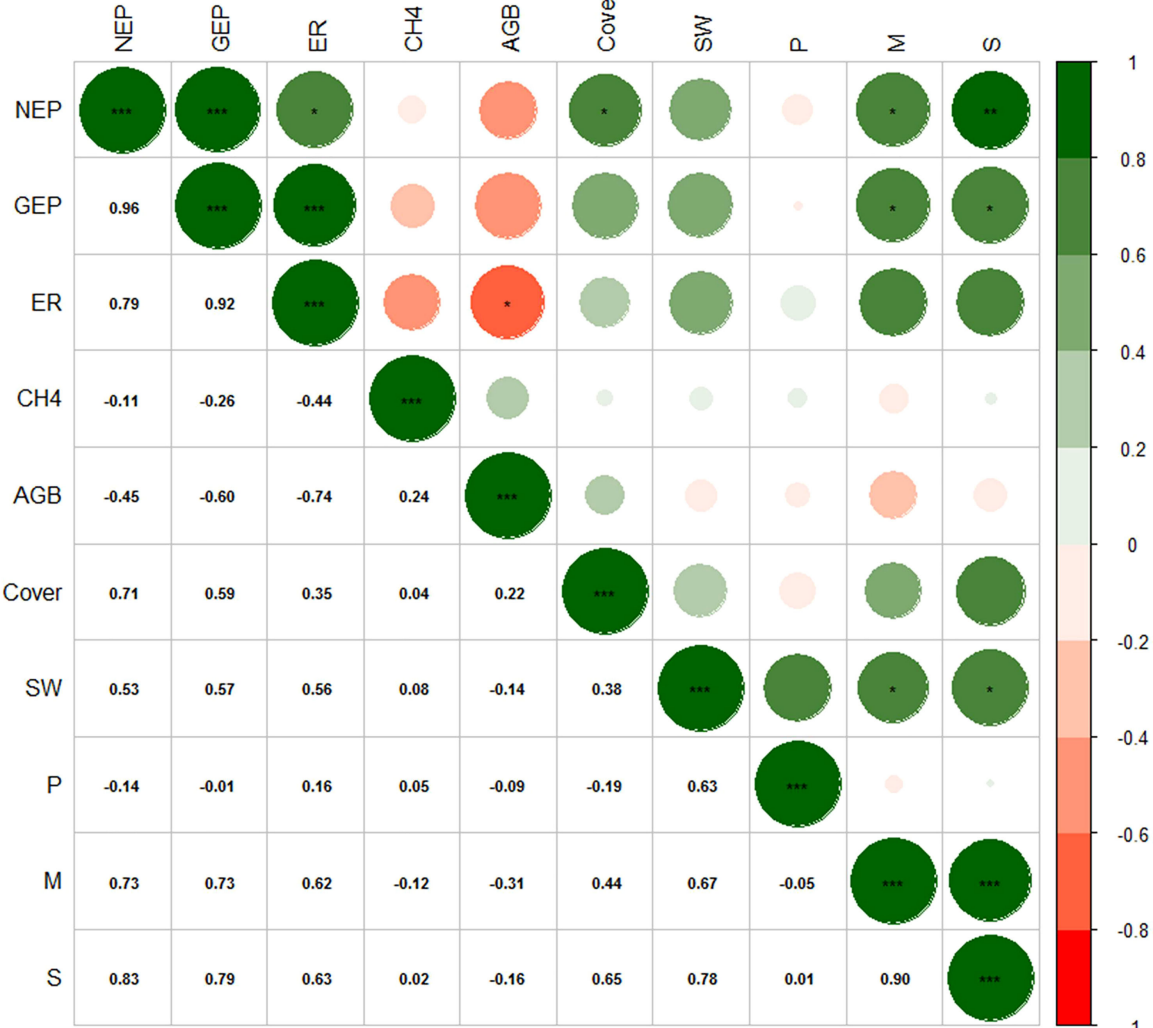
Heat map of the correlations between the vegetation characteristics and carbon fluxes. A green circle indicates that the correlation coefficient was positive and a red circle indicates that the correlation coefficient was negative. The larger the diameter of the circle, the higher the absolute value of the correlation coefficient. NEP: net ecosystem production, GEP: gross ecosystem production, ER: ecosystem respiration, CH_4_: methane fluxes, AGB: aboveground biomass, Cover: vegetation coverage, SW: Shannon-wiener index, P: Pielou index, M: Margalef index, S: number of species; “***”, “**” and “*” represent significant relationship of *P*< 0.001, *P*< 0.01 and *P*< 0.05, respectively.

The relationship between AGB and NEP in the alpine meadow was not significant. However, VC (R = 0.71, *P*< 0.05), species number (R = 0.83, *P*< 0.01), and the Margalef richness index (R = 0.73, *P*< 0.05) were positively correlated with NEP (**Figure 7**). The alpine meadow with more species and vegetation had a stronger carbon sink capacity. The species number (R = 0.79, *P*< 0.05) and the Margalef richness index (R = 0.73, *P*< 0.05) were also positively correlated with GEP, but VC and AGB had no significant relationship with GEP. ER in the alpine meadow was negatively correlated with AGB (R = −0.74, *P*< 0.05). Further analysis of the correlation between carbon fluxes and soil physiochemical characteristics showed that NEP and GEP were negatively correlated with the 0–5, 5–10, and 10–20 cm soil temperature values **(** **Figure S1** **)**. In addition, the relationships between carbon fluxes and SOC and TN were not significant. CH_4_ fluxes were not significantly related to vegetation or soil characteristics (**Figure S2**).

## 4 Discussion

Different grassland managements significantly affected carbon fluxes in the alpine meadow. Compared with free grazing and artificial grass planting, both GEP and ER increased significantly compared to enclosure, but the increase in GEP was greater than that in ER, so the carbon sink capacity of the alpine meadow increased significantly after enclosure. After 5 and 9 years of enclosure, NEP of the alpine meadow at Hongyuan increased significantly by 52% and 215%, respectively; and GEP increased by 55% after 9 years of enclosure (Du and Gao, 2021). Similarly, the photosynthetic capacity, AGB, GEP, and NEP of alpine meadow at the Qilian Mountains significantly increased after enclosure (Liu et al., 2019). In our study, ER of the artificial grassland was the lowest, and a study on Haibei alpine meadow also reported that ER of artificial grassland was lower, which was 1/2–1/3 of the WF grassland and natural grassland, respectively (Zhang Z. et al., 2018). Carbon fluxes of alpine meadow also affected by environmental factors. We found soil surface temperature had significant impact on GEP and NEP, which was consistent with previous studies (Hu et al., 2016; Yu et al., 2020). According to a four-year eddy covariance observation, soil water content was the main control on carbon fluxes of alpine meadow and will affect their response to temperature (Zhang T. et al., 2018). Furthermore, biodiversity not only affects the stability of grassland, but also affects the productivity (Grace et al., 2007; Yu et al., 2020). Biodiversity–productivity relationships vary with different plant functional group loss scenarios (Thakur et al., 2021; Pan et al., 2022). Due to the relatively single species composition of the artificial grass planting, GEP and NEP were significantly lower than enclosure plots. A study in the eastern Qilian Mountains showed that although the AGB of artificial grassland is significantly higher than that of an natural alpine meadow, the Shannon-Wiener diversity index and evenness index were significantly lower than those of the natural alpine meadow (Zhao et al., 2000). Although AGB of the artificial grassland in Henan County increased significantly after planting, no significant differences in species richness, the Shannon-Wiener diversity index, or the evenness index were observed (Zhang et al., 2020). However, the differences in the Shannon-Wiener diversity index and Pielou index of the alpine meadow under the three grassland managements were not significant in our study, but the number of species and the Margalef richness index of WF plots were significantly higher than those of the grazing and artificial planting plots, and the dominant community species were also different.

Alpine meadow ecosystems are complex, particularly the one at Maqin, which is an ecological barrier area located at the “Three River Sources”. It not only has a variety of ecological functions, such as acting as a carbon sink, but also has important production functions, such as animal husbandry and biomass production (Dong et al., 2020; Dong et al., 2022). Therefore, restoring and managing degraded alpine meadow should not be limited only to the recovery of vegetation or improving carbon sink capacity, but should comprehensively consider production and ecological functions to ensure sustainable development of the alpine meadow ecosystem (Yang M. et al., 2021). In our study, NEP and AGB of the artificial grassland was the highest, but the number of species and the richness index was lower. Therefore, enclosure is better than free grazing and artificial grass planting from the perspective of carbon sink capacity. A close-to-natural restoration theory was proposed based on the characteristics of the alpine ecosystem of the Qinghai-Tibetan Plateau, which includes the theories of biodiversity, ecosystem multi-functionality, and ecosystem stability [19]. This study also showed that NEP, which represented carbon sink capacity, was positively correlated with species composition and the richness index. Therefore, ecological stability should be considered when restoring alpine meadows. Native species should be the dominant species in a multi-species configuration of artificial grass plantings so that the community structure, species composition, and diversity of the degraded grassland are similar to local vegetation to achieve a variety of ecological and production functions in the alpine meadow.

The effects of different managements on carbon fluxes are complex and related to many factors, such as time of enclosure, condition of the grassland before enclosure, species composition for artificial grass planting, planting years, sowing method, and grazing regime (Xiang and Ma, 2021). A study of different durations of enclosure (2, 4, 9, 11, and 20 years) in Haibei alpine meadow reported that long-term enclosure reduces vegetation carbon storage by reducing root carbon storage, which is not conducive to root carbon sequestration (Song et al., 2020). Long-term enclosure (13 years) in Maqin County increases the density of soil seed banks but decreases species diversity (Huang, 2021). Species richness, community structure, and the succession stage of the artificial grassland varied with different planting years. Community structure and species diversity and richness generally affect carbon storage in artificial grassland ecosystems. Compared with 2-year and 5-year artificial grasslands, a 12-year artificial grassland had the highest species richness, which was gradually close to the natural grassland, and thus had a more stable community structure (Hu et al., 2016). Another 29-year long-term experiment in the central and northern United States showed that rotational grazing management increases soil carbon sequestration and stability compared with an artificial grassland (Rui et al., 2022). The results of a meta-analysis at the regional scale of the Qinghai-Tibetan Plateau revealed that the effects of grazing on vegetation and the soil carbon pool in the alpine grassland ecosystem are related to grazing intensity, as heavy grazing can lead to the degradation of grassland, whereas light grazing improves productivity (Yan et al., 2020). In this study, we tried to find a way to consider the restoration and carbon sink function of degraded grassland by comparing the carbon fluxes and their influencing factors under different grassland management types in an alpine meadow. However, more explanatory and comprehensive field studies considering the duration of the enclosure, species composition of the artificial grassland, and the grazing management method should be carried out to further explore the optimal management to restore and improve the carbon sink function of alpine meadows.

## 5 Conclusion

**Figure 8:**
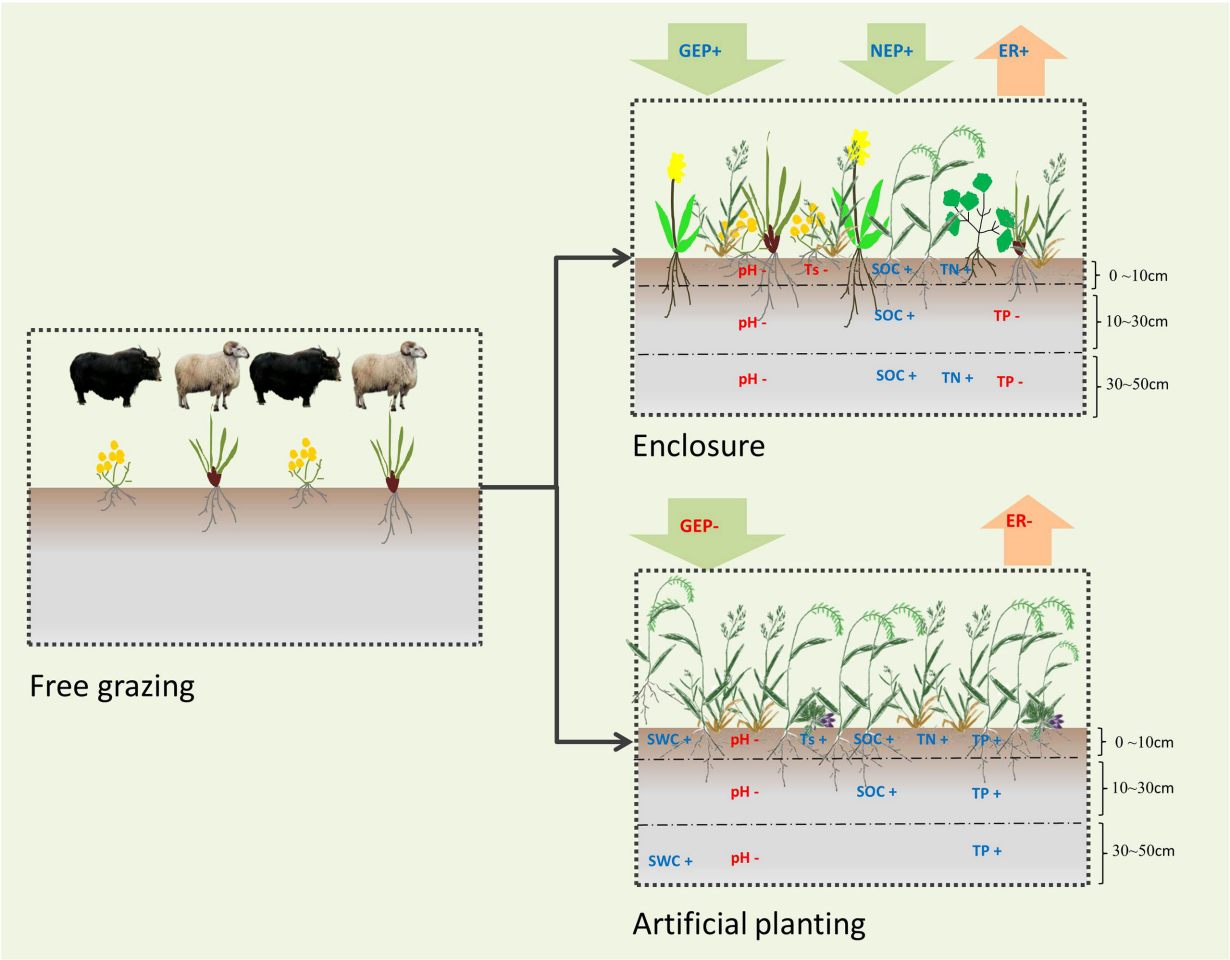
Concept map of different grassland managements on carbon fluxes, vegetation and soil characteristics of alpine meadow. NEP, net ecosystem production; GEP, gross ecosystem production; ER, ecosystem respiration; SWC, soil water content; Ts, soil temperature; SOC, soil organic carbon; TN, total nitrogen; TP, total phosphorus. For clarity, not all variables of our study are listed. Only the variables with significant changes compared with free grazing are listed. The blue word and red word indicates variables under the current management mode significantly increase or decrease compared with free grazing.

Alpine meadow in Maqin acts as a CO_2_ and CH_4_ sink. Grassland managements had significant effects on GEP, NEP and ER, but had no significant effect on CH_4_ fluxes. Artificial grassland had the highest aboveground biomass but enclosure grassland had the highest GEP and NEP (**Figure 8**). Furthermore, we found the number of species and the Margalef richness index of the alpine meadow community was positively correlated with GEP and NEP. Therefore, a multi-species configuration dominated by native species should be used to restore the degraded alpine meadow and improve carbon sink capacity while considering both grassland restoration and production functions.

## Data availability statement

The original contributions presented in the study are included in the article/**Supplementary Materials**. Further inquiries can be directed to the corresponding author.

## Author contributions

GX and XK contributed equally to this work. GX and XK conceived and designed the experiments. YC, YL, WL and SW conducted the experiments. LY, XZ, EK and ZY analyzed the data. LY and AY wrote the manuscript. XK, XW and LY revised the manuscript. All authors contributed to the article and approved the submitted version.

## Funding

This study was supported by Scientific and Technological Innovation Project of Northwest Surveying and Planning Institute of National Forestry and Grassland Administration (No. GLXD-2021-QT-20) and the National Natural Science Foundation of China (No. 42041005).

## Conflict of interest

The authors declare that the research was conducted in the absence of any commercial or financial relationships that could be construed as a potential conflict of interest.

## Publisher’s note

All claims expressed in this article are solely those of the authors and do not necessarily represent those of their affiliated organizations, or those of the publisher, the editors and the reviewers. Any product that may be evaluated in this article, or claim that may be made by its manufacturer, is not guaranteed or endorsed by the publisher.
