# Supplementary material for: Different grassland managements significantly change carbon fluxes in an alpine meadow
Source: Front Plant Sci. 2022 Oct 13;13:1000558. doi: 10.3389/fpls.2022.1000558 (PMC9606693; doi:10.3389/fpls.2022.1000558)
Supplement: Supplementary file 1 [file DataSheet_1.docx]

# Supplementary Material


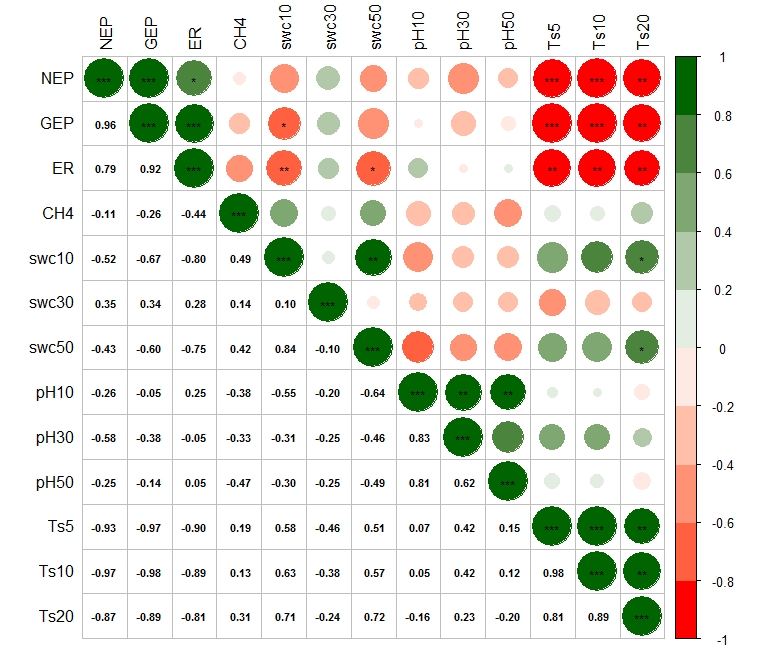


**Figure S1 Heat map of relationship between carbon fluxes and soil physical properties**. A green circle indicates that the correlation coefficient was positive and a red circle indicates that the correlation coefficient was negative. The larger the diameter of the circle, the higher the absolute value of the correlation coefficient. GEP: gross ecosystem production, ER: ecosystem respiration, CH_4_: methane fluxes, swc10: soil water content (0~10cm), swc30: soil water content (10~30cm), swc50: soil water content (30~50cm), pH 10: soil pH (0~10cm), pH 30: soil pH (10~30cm), pH 50: soil pH (30~50cm), Ts5: soil temperature (0~5cm), Ts10: soil temperature (0~10cm), Ts20: soil temperature (10~20cm); “***” , “**” and “*” represent significant relationship of *P* < 0.001, *P* < 0.01 and P < 0.05, respectively.


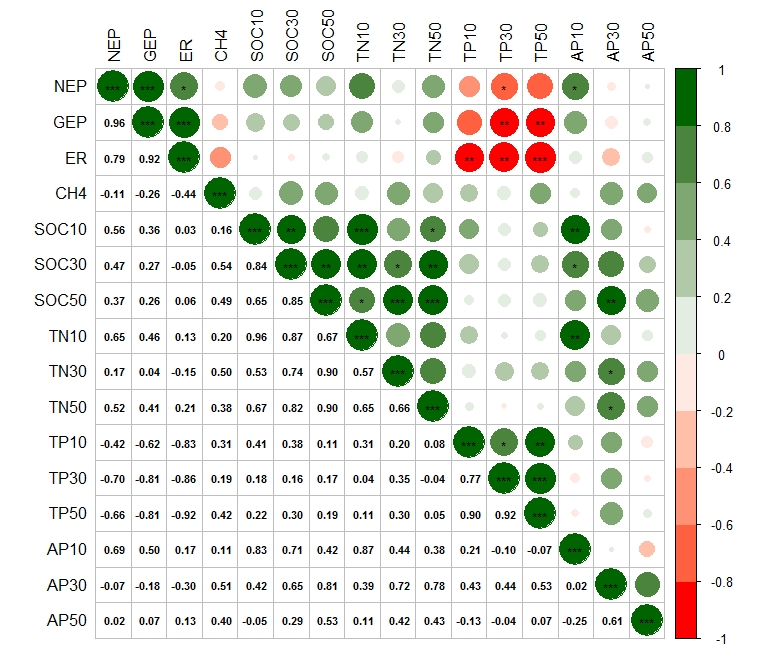


**Figgure S2 Heat map of relationship between carbon fluxes and soil C, N P content.** A green circle indicates that the correlation coefficient was positive and a red circle indicates that the correlation coefficient was negative. The larger the diameter of the circle, the higher the absolute value of the correlation coefficient. NEP: net ecosystem production, GEP: gross ecosystem production, ER: ecosystem respiration, CH_4_: methane fluxes, SOC10: soil organic carbon (0~10cm), SOC30: soil organic carbon (10~30cm), SOC50: soil organic carbon (30~50cm), TN 10: soil total nitrogen (0~10cm), TN 30: soil total nitrogen (10~30cm), TN 50: soil total nitrogen (30~50cm), TP 10: soil total phosphorus (0~10cm), TN 30: soil total phosphorus (10~30cm), TN 50: soil total phosphorus (30~50cm), AP 10: soil available phosphorus (0~10cm), AP 30: soil available phosphorus (10~30cm), AP 50: soil available phosphorus (30~50cm); “***” , “**” and “*” represent relationship of *P* < 0.001, *P* < 0.01 and P < 0.05, respectively.
